# Consensus design and engineering of an efficient and high-yield peptide asparaginyl ligase for protein cyclization and ligation

**DOI:** 10.1016/j.jbc.2023.102997

**Published:** 2023-02-09

**Authors:** Xinya Hemu, Xiaohong Zhang, Hong Yi Chang, Jin En Poh, James P. Tam

**Affiliations:** 1 School of Biological Sciences, Nanyang Technological University, Singapore, Singapore; 2 School of Traditional Chinese Pharmacy, China Pharmaceutical University, Nanjing, China; 3 Department of Pharmacy, Singapore General Hospital, Singapore, Singapore

**Keywords:** peptide asparaginyl ligase, asparaginyl endopeptidase, legumain, consensus design, β-ME, β-mercaptoethanol, AEP, asparaginyl endopeptidase, conLEG, consensus plant legumain, E:S, enzyme:substrate ratio, PAL, peptide asparaginyl ligase, SFTI, sunflower trypsin inhibitor, LAD, ligase activity determinant

## Abstract

Plant legumains are Asn/Asp-specific endopeptidases that have diverse functions in plants. Peptide asparaginyl ligases (PALs) are a special legumain subtype that primarily catalyze peptide bond formation rather than hydrolysis. PALs are versatile protein engineering tools but are rarely found in nature. To overcome this limitation, here we describe a two-step method to design and engineer a high-yield and efficient recombinant PAL based on commonly found asparaginyl endopeptidases. We first constructed a consensus sequence derived from 1500 plant legumains to design the evolutionarily stable legumain conLEG that could be produced in *E. coli* with 20-fold higher yield relative to that for natural legumains. We then applied the ligase-activity determinant hypothesis to exploit conserved residues in PAL substrate-binding pockets and convert conLEG into conPAL1–3. Functional studies showed that conLEG is primarily a hydrolase, whereas conPALs are ligases. Importantly, conPAL3 is a superefficient and broadly active PAL for protein cyclization and ligation.

Legumains are cysteine proteases that are widely distributed among plants and animals. These proteases were named after their discovery in legume seeds in the early 1990s (1). They have also been isolated from parasites (2) and mammals (3). Legumains belong to the same family of enzymes as vacuolar processing enzymes that were discovered in the late 1980s (4). Regardless of their origin, these enzymes share a similar protein fold and are classified in the C13 subfamily of Cys proteases (MEROPS, EC 3.4.22.34) (5). Functionally, legumains are asparaginyl endopeptidases (AEPs) that hydrolyze the peptide bond after an Asn/Asp(Asx) (Fig. 1). Mammalian legumains are lysosomal enzymes that play important roles in antigen presentation and cancer-related events (6). In contrast to animal genomes that have only one gene encoding a legumain, plant genomes contain multiple copies of legumains. The major functions of plant legumains are to regulate senescence processes (7), maturation of seed storage proteins (8), and processing of defensive proteins and peptides.

**Figure 1:**
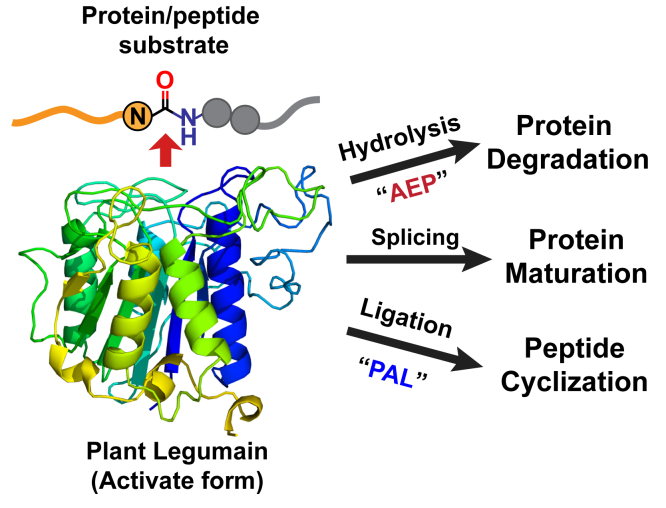
**Schematic illustration of enzymatic activity and biological functions of plant legumain**.

In addition to cutting peptides at specific Asx sites, legumains can also join peptides (9). An early example of this activity was observed in the maturation of the legume lectin, concanavalin A (conA), which involves a splicing (cut-and-join) mechanism. This protease-mediated peptide ligation is assisted by the correctly folded conformation of the conA precursor that brings two ligating termini in close proximity to facilitate a transpeptidation reaction (10). Recently, we identified a subtype of plant legumains that act as ligases rather than proteases. We termed these enzymes peptide asparaginyl ligases (PALs). Examples of PALs include the prototype butelase-1 from *Clitoria ternatea* (11), which has a catalytic efficiency of 1.3 × 10^6^ M^−1^s^−1^, making it the fastest natural peptide ligase for peptide macrocyclization (12). Other PALs include OaAEP1b from *Oldenlandia affinis* (13, 14) and a dozen more from *Viola* plants (15, 16, 17). Compared with widely distributed AEPs in both primitive and higher plants, PALs in nature are much rarer, only accounting for 1% of sequenced plant legumains (18).

Butelase-1 and other PALs serve as bioprocessing enzymes of macrocyclic peptides such as cyclotides and trypsin inhibitors (11, 13, 15, 19, 20, 21). They recognize an Asx-Xaa-Yaa tripeptide motif in which Xaa represents any residue except Pro and Yaa represents hydrophobic residues (11, 15). PALs function as efficient, site-specific ligases that do not require ATP for activity. As such, they are invaluable for biochemical and pharmaceutical applications for protein engineering and site-specific modifications of proteins and live cells (22, 23, 24, 25, 26, 27, 28).

A challenge to the wider use of PALs in industrial applications is associated with their recombinant productions. Recombinant PAL proenzymes can be expressed in bacteria, but the expressed proteins are often present as misfolded proteins in inclusion bodies (11) and the yield of soluble proteins is fairly low (29). Although incorporation of large fusion tags such as maltose-binding protein can increase the yield of crude fusion proteins, they can also lead to heterogeneous activity of the enzymes after activation (30, 31). Secretory expression in insect cells could produce about 20 mg/L of VyPAL2, but this approach is limited by relatively high costs. Similarly, secretory expression of C-terminal truncated butelase-1 in yeast yielded 16 mg/L active enzyme (32), but the success of this method has yet to be repeated for production of other PALs. Attempts to improve expression levels using disulfide-promoting strains, codon modification, truncation of the flexible N-terminal proregion, and incorporation of fusion tags suggest that expression of soluble PALs could be facilitated by increasing the stability of cDNA, mRNA, or peptide chains (28). By using residues that appear most frequently among homologous genes, an engineered functional protein could acquire enhanced stability, or activity, or both (33, 34, 35, 36, 37).

Here, we report a strategy to design high-yield PALs. We developed this strategy using bioinformatic analysis of a dataset containing 1500 plant legumains to build a consensus legumain followed by mutation of substrate-binding pocket residues based on the ligase-activity determinants (LAD) hypothesis that was previously developed to convert an AEP to a PAL.

## Results

### Consensus plant legumain sequence for recombinant protein expression

**Figure 2:**
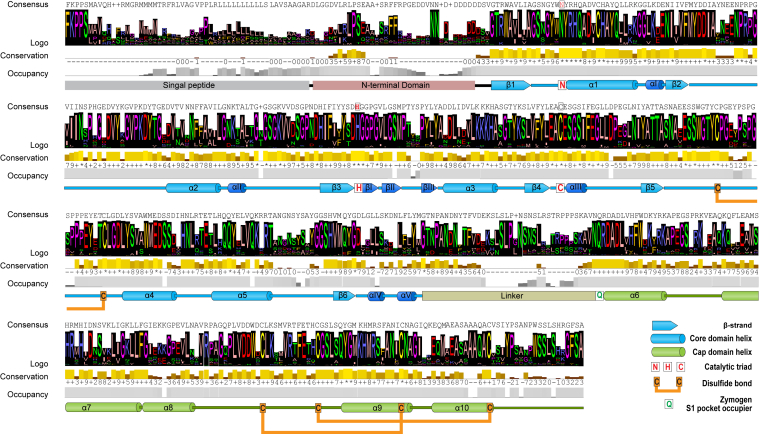
**Design of the consensus plant legumain sequence based on multiple sequence alignment and analysis of 1500 plant legumains.** The secondary structures, protein domains, and functional features were annotated based on crystal structure of AtLEGβ (Protein Data Bank code: 5NIJ).

As recently reported, a dataset containing 1500 plant legumains from 249 plant families was constructed based on BLAST searches of existing protein sequences and transcriptomes in NCBI and 10KP databases, respectively, using representative AEPs and PALs as queries (18). Multiple sequence alignment of these legumains produced a consensus sequence having a typical protein fold of C13 Cys proteases, including an α5β6 core domain carrying the “Asn-His-Cys” catalytic triad and a five-helix cap domain (38) (Fig. 2).

**Figure 3:**
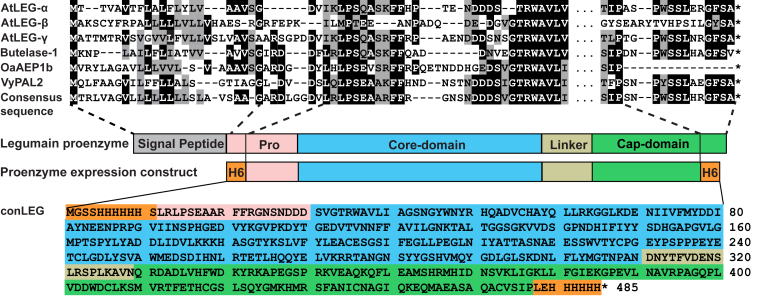
**Construction of conLEG sequence for recombinant expression.** The construct encoded full-length proenzyme derived from the consensus sequence of 1500 plant legumains, with a His6-tag attached to the N terminus of the conserved region of the prodomain.

The expression construct consensus plant legumain (conLEG) was designed by removing residues with <10% occupancy in the 1500 plant legumains, substituting the signal peptide and the nonconserved region of the prodomain with a His6 affinity tag, and substituting the C-terminal unstructured loop after helix α10-Pro457 (corresponding to butelase-1 Pro469) with a C-terminal His6-tag (Figs. 3 and S1*A*).

Recombinant protein was expressed from the conLEG construct as a proenzyme in *E. coli* Shuffle-T7B. After 48 h induction, 36 to 40 mg/L purified protein was obtained (Fig. S1*B*). Acid-induced autoactivation of conLEG was carried out under different pHs and temperatures. The optimized conditions for autoactivation were acidification of a 1 mg/ml proenzyme solution to pH 4.0 and incubation for 2 h at 37 °C (Fig. S1*C*). After size exclusion chromatography purification, a final yield of 12 mg active enzyme per 1 L culture was obtained.

### conLEG functions mainly as a protease but has cyclase and splicing activity toward some substrates

**Figure 4:**
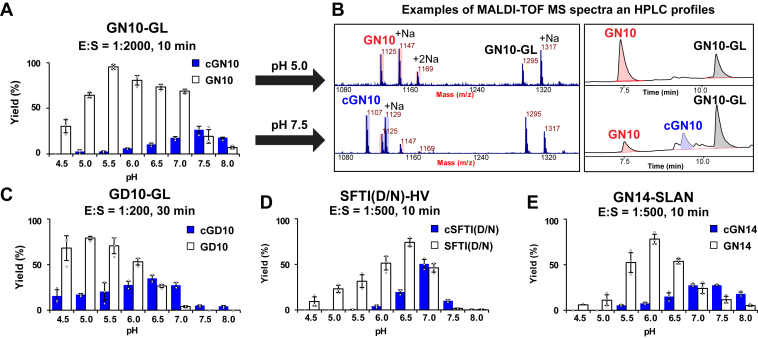
**conLEG-mediated hydrolysis and cyclization of peptide substrates.** Quantitative product distribution of (*A*) GN10-GL and its representative examples of mass spectrometry and HPLC data at pH 5 and pH 7.5 in (*B*), (*C*) GD10-GL, (*D*) SFTI(D/N)-HV, and (*E*) GN14-SLAN. Reactions were carried out at 25 °C in triplicate and monitored by MALDI-TOF mass spectrometry. Quantified data by reverse-phase HPLC are presented in column plots. E:S ratio and reaction time are indicated in each plot.

To test the innate enzymatic ability of a plant legumain to catalyze hydrolysis, splicing, and ligation, we carried out a functional study of activated conLEG toward four synthetic peptide substrates, including a pair of short peptides GN10-GL (GISYKPAYLNGL, MW 1295 Da) and GD10-GL (GISYKPAYLDGL, MW 1296 Da), the cyclotide-mimicking GN14-SLAN (GISTKSIPPISYRNSLAN, MW 1918 Da), and sunflower trypsin inhibitor (SFTI) (D/N)-HV (GRCTKSIPPICFPNHV, MW 1768 Da) that mimics the precursor of the cyclic trypsin inhibitor SFTI-1 (Fig. 4). Reactions were performed with the same substrate concentration of 20 μM at eight reaction pHs ranging from 4.5 to 8.0 at 25 °C. Since the catalytic rate was substrate dependent, different enzyme:substrate (E:S) ratios and reaction times were set for each substrate to prevent overreaction. Overall, GN10-GL is the most favored substrate of conLEG with an E:S ratio of 1:2000 and 10 min reaction time, and GD10-GL is the least favored substrate with an E:S ratio of 1:200 and 30 min reaction time. SFTI(D/N)-HV and GN14-SLAN were reacted for 10 min with an E:S ratio of 1:500.

At acidic pH, conLEG acts as an AEP and hydrolyzed all four peptide substrates as monitored by HPLC. At neutral and slightly basic pH, conLEG displayed low ligase activity (Fig. 4*A*). For example, at pH 5, >60% of the 12-residue peptide GN10-GL was hydrolyzed to the 10-residue GN10 (GISYKPAYLN, MW, 1125 Da) with <3% cGN10 (cyclo-GISYKPAYLN, MW, 1107 Da), resulting in a very low cyclization/hydrolysis (C/H) ratio of 0.04 (Fig. 4*B*). In contrast, at pH 7.5, 19% of GN10-GL was hydrolyzed and 26% was cyclized, yielding a C/H ratio of 1.4. Similar product distribution profiles in which the C/H ratio increased with pH were obtained for the other three substrates (Fig. 4, *C*–*E*).

We also compared the catalytic effect of conLEG on two different peptide substrates: GN10-GL and GD10-GL, which have an Asn and Asp at the P1 recognition site and P1 position, respectively. P1-Asp resulted in higher C/H ratios at all eight pH values tested (Fig. 4*C*). The generality of cyclic product formation in conLEG-mediated reactions suggest that plant legumains function intrinsically as AEPs under acidic pH conditions but the activity can also be bidirectional and influenced by extrinsic factors such as pH as well as by substrate and sequence.

**Figure 5:**
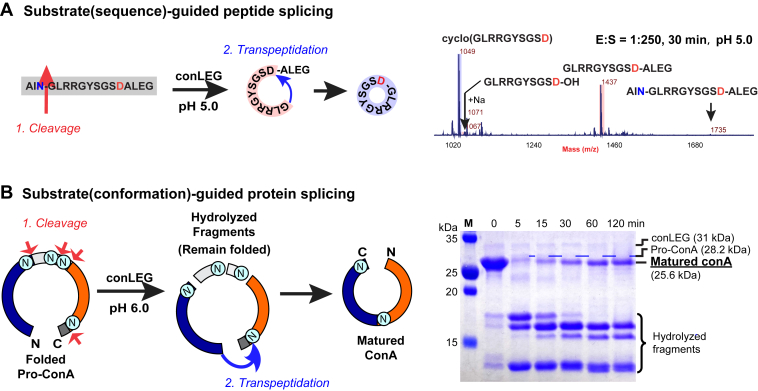
**conLEG-mediated Asn/Asp-specific cleavage and transpeptidation**. *A*, conLEG-mediated N-terminal Asn-specific hydrolysis and C-terminal Asp-specific transpeptidation of AINGLRRGYSGSDSLEG to produce cyclo(GLRRGYSGSD). The reaction was carried out at pH 5.0 in an E:S ratio of 1:250 at 37 °C for 30 min to facilitate release of the N-terminal tripeptide cap AIN. *B*, conLEG-mediated maturation of conA by Asn-specific hydrolysis followed by structural-guided transpeptidation.

To determine whether conLEG can act as a splicing enzyme when Asp instead of Asn is present at the P1 position, we synthesized a model substrate AINGLRRGYSGSDALEG (1735 Da) containing both Asn and Asp to mimic the precursor of Momordica cochinchinensis trypsin inhibitor (McoTI)-II (21, 39). At pH 5.0, we observed formation of the Asn-cleaved intermediate GLRRGYSGSD-ALEG (1437 Da) and a cyclic product cyclo-(GLRRGYSGSD) (1049 Da), suggesting that conLEG could catalyze both Asn-specific hydrolysis and Asp-specific transpeptidation under acidic pH (Fig. 5*A*). A similar trimodal enzymatic activity was reported for McPAL1, the bioprocessing enzyme that mediates cyclization of McoTI-II at Asp *via* a splicing mechanism (21).

To show conLEG splicing activity toward proteins, we recombinantly expressed a protein substrate, concanavalin A precursor (pro-conA), which was recombinantly expressed, folded by dialysis, and then matured by conLEG-mediated processing (Fig. S2). Monitoring of conLEG splicing at different time points over 2 h using SDS-PAGE showed that folded pro-conA (28.2 kDa) was rapidly cleaved into smaller fragments within 5 min followed by gradual accumulation of mature conA (25.6 kDa) over time (Fig. 5*B*). These results were similar to those reported for conA maturation with its native bioprocessing enzyme CeAEP1 (10). Of note, the *in vitro* splicing reaction or transpeptidation reaction was slow and took hours to reach completion that accompanied with high hydrolysis.

### Conversion of conLEG to conPAL1–3 by mutation of LAD residues in PAL substrate-binding pockets

**Figure 6:**
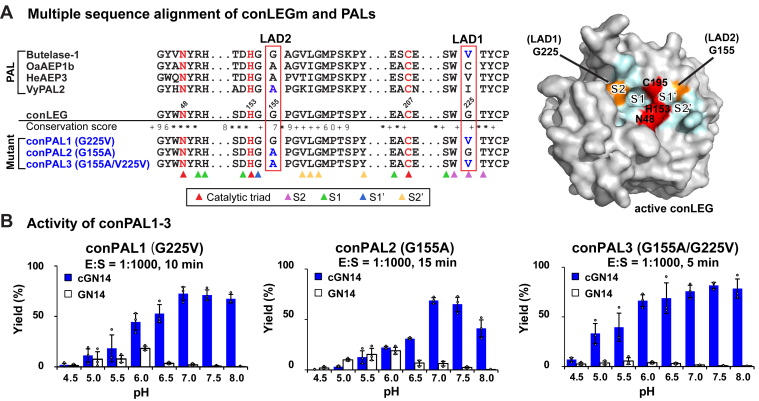
**Peptide asparaginyl ligase (PAL)-like conLEG mutants based on ligase-activity determinant (LAD) hypothesis.***A*, location of the key LAD residues G225 and G155 in the conLEG and PAL sequence alignment and conLEG model structure. The catalytic triad N-H-C is in *red*. LAD mutations in conPAL1–3 are *highlighted in blue and orange* in the model structure. Substrate-binding residues S2-S1-S1′-S2′ are indicated by different arrows and colored cyan in the structure. *B*, activity screening of conPAL1–3 at different pHs with GN14-SLAN as the substrate. Reactions were performed in triplicate in an E:S ratio of 1:1000 at 25 °C and quantified with reverse-phase HPLC.

To convert conLEG to a PAL, we targeted LADs in substrate-binding pockets that determine whether the legumain activity is primarily AEP or PAL (15, 40). In PALs, such as butelase-1 and VyPAL-2, the critical direction-steering LAD motifs have hydrophobic residues substituted for two conserved Gly residues that flank the S1 catalytic center (11, 15). In conLEG, LAD1 and LAD2 have Gly225 and Gly155, respectively, which are both AEP-like. To convert conLEG into PAL-like ligases, we made a Gly225Val mutation in LAD1 to generate conPAL1 that mimics butelase-1 and a mutation Gly155Ala in LAD2 to generate conPAL2 that mimics VyPAL2 (Fig. 6*A*). Finally, we engineered conPAL3, having a G155A/G225V double mutation in the substrate-binding pockets, which combines features of the two single mutants conPAL1 (butelase-1-like) and conPAL2 (VyPAL-like).

Recombinant conPAL1–3 were expressed using the same protocol as for conLEG, and their enzymatic activity was examined by MALDI-TOF mass spectrometry at 25 °C with the substrate GN14-SLAN at a fixed molar ratio of E:S = 1:1000 and at eight different pHs, ranging from 4.5 to 8.0 (Figs. 6*B* and S3). In contrast to the AEP-like conLEG, all three conPALs displayed predominant ligase activity. In particular, the double mutant conPAL3 had a C/H ratio >20 (equivalent to >95% yield) at pH ≥6.5.

### conPAL3 displays high catalytic efficiency

**Figure 7:**
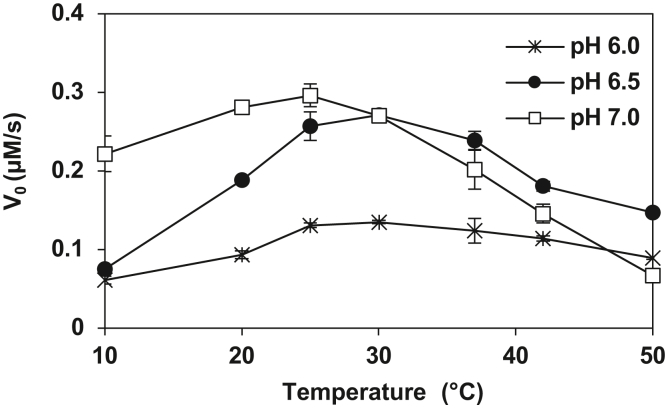
**Optimal pH and temperature of conPAL3-mediated GN10-GL cyclization.** Reactions were performed in triplicates at seven different temperatures and three different pH values with a 1:10,000 E:S ratio. To calculate the initial catalytic rates, aliquots of the reaction mixtures were collected and quenched every 30 s for quantitative analysis by reverse-phase HPLC.

We further characterized conPAL3 for its substrate specificity, catalytic efficiency, and stability. We first determined the optimal reaction conditions for conPAL3 by examining the initial catalytic rate of GN10-GL cyclization at three different pHs (6.0, 6.5, and 7.0) and seven different temperatures (10, 20, 25, 30, 37, 42, and 50 °C) (Fig. 7). The conPAL3-mediated reactions had an optimal pH of 7.0, and the optimal reaction temperature was 25 °C. This result differs slightly from natural PALs like butelase-1 or VyPAL2, which have an optimal reaction temperature between 37 °C and 42 °C and an optimal pH of 6 to 6.5.

The amino acid preference of substrate-binding pockets S2-S1-S1′-S2′ was screened using panels of synthetic substrates having Asn or Asp at the P1 position and saturated variation at the P2, P1′, P2′, P1”, or P2” positions (Table 1 and Fig. S4). The screening indicated that the conPAL3 S1 pocket favors Asn over Asp (Fig. S4*A*). The catalytic efficiency against GN10-GL was at least 80-fold higher (*k*_cat_/K_m_ = 518,031 M^−1^s^−1^ at the optimum pH 7.0) relative to GD10-GL (*k*_cat_/K_m_ = 6431 M^−1^s^−1^ at the optimum pH 5.0) (Fig. S5, *A* and *B*). The S2 pocket accepted any amino acid except the conformationally restricted Pro (Fig. S4*B*). The S1′ pocket is the binding site for both the leaving group P1′ residue and the P1” residue from the incoming nucleophile. S1′ had broad amino acid tolerance to P1′ but was less tolerant to changes at the incoming P1”. Gly was the most favored P1” residue (Fig. S4*C*). The S2’’ pocket, which also binds both P2′ and P2’’ residues, favored hydrophobic residues like Phe, Leu, Ile, and Met. This pocket is more tolerant to variations at P2′ than at incoming P2’’. Overall, conPAL3 has similar substrate preference to PALs but the less favorable leaving groups and incoming groups increased the likelihood of hydrolysis. Based on the results of the substrate specificity screening, a model substrate GFSYKPAYSN-GI (MW 1303.44 Da) was designed and synthesized. At the optimum pH 7.0, catalytic efficiency of conPAL3 toward this model substrate was highly efficient at 2,289,260 M^−1^s^−1^, which is nearly 2-fold faster than the reported efficiency for butelase-1 (Table 1 and Fig. S5*C*).

**Table 1:** Enzymatic and chemical properties of conPAL3

**A. Substrate specificity**			

**Pocket**	**Site**	**Substrate**	**Preference**

S1	P1	GISYKPAYL**N**GL	N>>D
		GISYKPAYL**D**GL	
S2	P2	GISTKSIPPISY**X**NGI	Any AA except P
S1′	P1′	GLYRRGRLYRRN**X**L	Any AA
	P1”	**X**LYRRLYRLNGI	Favor G
		**X**RLYRGRLYRRNHV	
S2′	P2′	GLYRRGRLYRRNG**X**	Any AA
	P2”	G**X**LYRGRLYRRNHV	Favor F, I, M, L
		R**X**ARRLYRLNGI	

**B. Enzyme kinetics**				

**Substrate**	**pH**	**kcat (s**^**−1**^**)**	**K**_**M**_**(μM)**	**kcat/Km (M**^**−1**^**s**^**−1**^**)**

G**I**SYKPAYL**N**-GL	7.0	16.52	31.89	518,031
G**I**SYKPAYL**D**-GL	5.0	0.32	49.54	6431
G**F**SYKPAY**SN**-GI	7.0	13.28	5.80	2,289,260


**C. Tolerance against nonaqueous solvents and adducts**		

**Solvent**		**Tolerance (%)**

Polar protic (alcohol)	Methanol	40.0
	Ethanol	30.0
	*iso*-Propanol	10.0
Polar aprotic	Acetonitrile	50.0
	Dimethyl sulfoxide	20.0
	Dimethyl formamide	10.0
Surfactant	Tween-20	2.0
	SDS	0.1

X, 20 natural amino acids. Letter in bold and underlined, site with a differential residue.

The tolerance of conPAL3 for nonaqueous solvents was examined by activity tests performed with various concentrations of polar protic solvents, polar aprotic solvents, and surfactants. conPAL3 retained 90% activity in the presence of 50% acetonitrile, 40% MeOH, 30% EtOH10%, iPrOH30%, dimethyl sulfoxide, 10% dimethyl formamide, and 2% Tween-20 but was rapidly inactivated by 0.1% SDS (Table 1C and Fig. S6).

## Discussion

**Figure 8:**
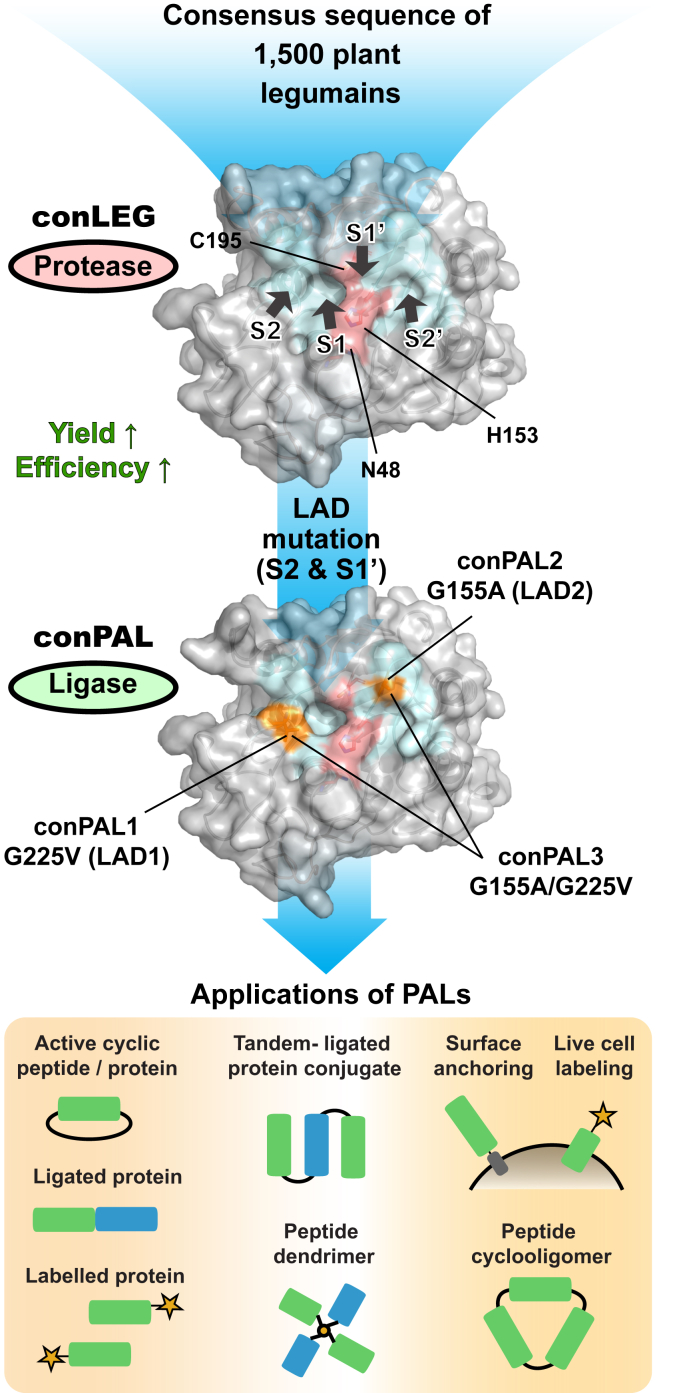
**Summary of consensus design and ligase-activity determinant (LAD)-based engineering of high-yield and efficient peptide asparaginyl ligases (PALs) as versatile tools for peptide and protein engineering**.

In this study, we combined a consensus AEP sequence found in 1500 legumains with conserved LADs found in the substrate-binding sites of a small group of known PALs to generate new PALs having significantly improved biochemical properties. The direct use of a consensus sequence derived from a large set of plant legumain sequences reduced the phylogenetic bias. Compared with natural legumains expressed in the most economically efficient bacterial system, we observed a 20-fold increase in conLEG expression, from an average of 2 mg/L proenzyme to about 40 mg/L. This increase may be due in part to improved mRNA stability and peptide solubility that facilitates translation and folding processes (41). Since the discovery of butelase-1 in 2014, we and others have unambiguously demonstrated PALs for cyclization of peptides and proteins, site-specific modifications of proteins and cell surfaces (Fig. 8) (22, 23, 24, 25, 26, 27, 28, 42, 43). Ligation at Asx is a challenge in peptide synthesis due to the unstable Asx-ester/thioester intermediates that are prone to the formation of aspartimide or succinic anhydride (44). Asx-specific PALs fill this role with high efficiency. An interesting outcome of this work is that the new PALs are tolerant to some organic reaction conditions that render them suitable to prepare dendrimers, polymers, and peptide–organic molecule conjugates as probes and for drug development. This high-yield and efficient conPAL3 could serve as a new model for further engineering and applications of PALs.

The designed conLEG shares 64 to 88% core-domain identity with 20 previously described AEPs and PALs (Fig. S7). The consensus design described here also improved the activity of plant legumains. The double-mutant conPAL3 carrying LAD mutations on both sides (S2, and S1′-S2′) of the S1 Asx-binding pocket exhibited similar catalytic efficiency toward a model peptide as the fastest natural PAL, butelase-1. In addition, conPAL3 has broad substrate specificity toward P1-flanking sequences and optimal ligase activity at neutral pH and ambient temperature, as well as a wide tolerance to various nonaqueous solvents. We also observed that the reaction pH affects the thermal stability of conLEG, suggesting that the stability of its active conformation is closely associated with intramolecular charge–charge interactions. Together, our results demonstrate that a consensus design based on a large sequence dataset is a feasible approach to provide improved functional proteins.

Protease activity was the first identified and most frequently observed property of legumains. As such, the term AEP is often used interchangeably with legumain as a representative of the C13 subfamily. Our work used an “averaged” sequence to show that plant legumains primarily act as hydrolases, particularly under acidic conditions, but they can be conditionally bidirectional in rare cases, particularly serving as bioprocessors of certain peptides and proteins as well as during seed germination of seed storage proteins. Our results show that the activity of plant legumain is influenced by environmental factors such as pH, substrate composition (such as P1-Asp), and conformation (such as folded conA) or by intrinsic factors such as LAD mutations that led to the emergence of PALs. Most proteolytic enzymes appear to be unidirectional in nature, likely due to the lack of a ligation-promoting environment or the absence of a coevolved substrate for ligation. We speculate that both intrinsic and extrinsic factors contribute to the ligase activity of AEPs and PALs *via* a general mechanism: a favored coordination of substrates in the catalytic center that increases the thioester intermediate stability and inhibits the access of catalytic water by retaining the prime-side leaving groups in pockets (15, 21, 40). This mechanism could be applied to explain other protease-based engineering of ligases, such as the subtilisin-BPN’-derived subtiligase and its kinds (45, 46, 47), and trypsin-derived tryptiligase that specifically recognizes a YRH motif in its substrates (48). Based on comparison of experimental results, we conclude that the selected intrinsic and extrinsic factors could exert profound influence on the enzymatic directionality toward AEPs or PALs, which may enlighten the design of other protease-derived ligases.

## Experimental Procedures

### Generation of plant legumain consensus sequence

Full amino acid sequences of butelase-1, OaAEP1b, AtLEGα-γ, and butelase-2 were used as representative plant PALs and AEPs to search homologous sequences in NCBI (BLASTp—nonredundant protein [nr] database, tBLASTx—nr and Transcriptome Shotgun Assembly database) and 10KP database. The search yielded 1500 nonduplicated hits as reported recently (18). Multiple sequence alignment was performed using the ClustalOmega program and analyzed by Jalview to generate visualization data including sequence Logo, consensus sequence, conservation, and occupancy. Regions with <10% occupancy were removed from the consensus sequence.

### Plasmid construction and site-directed mutagenesis

The conLEG cDNA sequence was codon-optimized, synthesized, and subcloned into the pET28a(+) vector with restriction cleavage by NdeI/XhoI to carry both N- and C-terminal His6 tags (GenScript). Primers carrying a G155A or G225V mutation were designed for site-directed mutagenesis using a Q5 Site-Directed Mutagenesis Kit (New England Biolabs) to generate conPAL1–3 plasmids.

### Recombinant expression of conLEG and mutants

Plasmids were transformed into SHuffle T7 *E. coli* competent cells (NEB, C3029J). LB broth (kanamycin+) cultures (1 L) were grown at 30 °C with shaking at 180 rpm until the *A*_600_ reached 0.4. The cultures were cooled to 16 °C before isopropyl β-D-1-thiogalactopyranoside (IPTG, 0.1 mM) was added, and the cultures were further incubated for 24 to 48 h. Cell pellets were harvested by centrifugation at 6000*g* for 15 min at 4 °C and resuspended in cold lysis buffer (50 mM Na Hepes, 0.1 M NaCl, 1 mM EDTA, 5 mM β-mercaptoethanol [β-ME], 0.1% TritonX-100, pH 7.5) in a ratio of 20 ml per 1 g pellet. Cells were lysed by sonication (10 min for 50 ml, 2s on/8s off) on ice. Clarified cell lysates were obtained by centrifugation at 12,000*g* for 15 min and subjected to affinity purification on a 5-ml Excel HisTrap affinity purification column (GE Life Sciences) equilibrated with IMAC-A buffer (20 mM sodium phosphate buffer pH 7.5, 0.1 M NaCl, 5% glycerol, 1 mM EDTA, 5 mM β-ME). The column was then washed with a mixture of IMAC-A and IMAC-B (0.5 M imidazole in buffer IMAC-A, pH 7.5) buffer containing 0.25 M imidazole (95/5 v/v, IMAC-A/IMAC-B). Targeted proteins were eluted with IMAC-B buffer containing 0.5 M imidazole. Fractions containing the targeted proenzymes were further purified on a size exclusion chromatography column (HiLoad 16/600 Superdex 200, Cytiva) equilibrated with SEC-7.5 buffer (20 mM sodium phosphate buffer pH 7.5, 0.1 M NaCl, 5% glycerol, 1 mM EDTA, 5 mM β-ME) to remove imidazole.

### Activation of conLEG and mutants

Acid-induced enzyme activation was performed at pH 3.5 to 6.0. Optimal activation was observed with pH 4.0 to 4.2 and incubation for 2 to 2.5 h at 37 °C with approximately 1 mg/ml proenzyme in 20 mM sodium citrate buffer containing 5 mM β-ME and 1 mM EDTA. Activated enzymes were purified by size exclusion chromatography equilibrated with SEC-4 buffer (20 mM sodium citrate buffer, 5 mM β-ME, 5% glycerol, 0.1 M NaCl, pH 4.0).Fractions containing target proteins were neutralized with storage buffer (20 mM sodium citrate, 5 mM β-ME,1 mM EDTA, 20% sucrose, 0.1 M NaCl, pH 5.5) and kept at 4 °C or stored at −80 °C after rapid freezing in liquid nitrogen.

### Preparation of peptide substrates

Peptide substrates (GN10-GL, GD10-GL, GN14-SLAN, SFTI(D/N)-HV, GFSYKPAYSN-GI) were synthesized by Fmoc chemistry using an automated microwave synthesizer (Liberty Blue, CEM) and purified by preparative reverse-phase HPLC (LC-20A, Shimadzu). Peptide substrate libraries (GISTKSIPPISYXNGI, XLYRRLYRLNGI, RXARRLYRLNGI, GLYRRGRLYRRNXL, XRLYRGRLYRRN-HV, GLYRRGRLYRRNGX, GLYRGRLYRRNHV, X = 20 natural amino acids) were purchased from GL Biochem.

### Preparation of recombinant pro-conA and conLEG-mediated maturation of conA

cDNA encoding the His6-TEV-pro-conA sequence was synthesized and subcloned into pET28a(+) in frame with the N-terminal His-tag (GenScript), followed by transformation into Rosetta PLysS competent cells (Novagen). The overnight culture was amplified from 1 ml to 1 L with LB broth at 37 °C with shaking at 180 rpm until the *A*_600_ reached 0.5. Recombinant expression of pro-conA was induced by addition of 0.5 mM IPTG and incubation for 24 h at 16 °C with shaking at 180 rpm. The cell pellet was harvested by centrifugation at 6000*g* for 10 min at 4 °C. The pellets were resuspended in 10× w/v conA lysis buffer (20 mM Mops, 0.5 M NaCl, 1 mM CaCl_2_, 1 mM MnCl_2_, 0.1% Triton X-100, pH 7). After 5 min sonication on ice, inclusion bodies were collected by centrifugation at 12,000*g* for 15 min at 4 °C. The pellets were washed twice with conA buffer (conA lysis buffer without Triton X-100). Purified inclusion bodies were resuspended in 40 ml refolding solution (conA buffer with 6 M guanidine-HCl, pH 7) followed by 1 min sonication. The soluble portion was isolated by centrifugation at 12,000*g* for 30 min at 4 °C. Recombinant protein was refolded by gradual dilution with 30× conA buffer followed by purification using size exclusion chromatography. To remove the N-terminal tag, TEV protease was mixed with recombinant His6-TEV-pro-conA at a 1:10 w/w ratio and incubated at 25 °C for 4 h or 4 °C overnight. Pro-conA was purified from the TEV protease by reverse IMAC. For conLEG-mediated circular permutation, pro-conA was buffer-exchanged with legumain reaction buffer (20 mM sodium phosphate, 5 mM β-ME, 1 mM EDTA, pH 6.0) using a centrifugal concentrator (Vivaspin Turbo 15, Sartorius) and concentrated to a final concentration of ∼1 mg/ml (calculated molar concentration 30 μM based on 280 nm absorbance). Active conLEG was added to the conA solution to reach a molar ratio of conLEG:pro-conA = 1:100. Reactions were performed at pH 6.0 at 25 °C. Reaction mixtures were analyzed by SDS-PAGE at 0, 5, 15, 30, 60, and 120 min.

### Functional studies of active conLEG and conPAL1–3 mutants

Functional studies of conLEG and mutants were carried out with synthetic peptide substrates and peptide libraries. The concentration of peptide substrates was fixed at 20 μM unless specifically mentioned. All reactions were monitored by MALDI-TOF mass spectrometry (5800 TOF/TOF, Applied Biosystem) and if needed, quantitatively analyzed by reverse-phase HPLC on a C18 analytical column (Aeris WIDEPORE, Phenomenex) after quenching with 1:1 v/v acetonitrile containing 0.1% trifluoroacetic acid.

Reactions in Figure 4 were performed with conLEG at 25 °C at eight pHs ranging from 4.5 to 8.0 in 20 mM citrate or phosphate buffers with 1 mM EDTA and 5 mM β-ME. Since the reaction rate was substrate dependent, different amounts of conLEG were added to give a specific E:S ratio ranging from 1:200 to 1:2000. Reaction times were also set differently as indicated in the figure legends and plots. Reactions in Figure 5*A* were performed at 37 °C at pH 5.0 for 30 min in an E:S ratio of 1:250. Reactions in Figure 6*B* were performed with conLPAL1-3 in an E:S ratio of 1:1000 at 25 °C in eight pHs ranging from 4.5 to 8.0 for 10, 15, and 5 min, respectively. Substrate specificity study in Fig. S4 was performed in different E:S ratios ranging from 1:100 to 1:2000 at 25 °C for 5 to 30 min at pH 7.0. Detailed conditions for each set of substrates were given in the data plot and figure legend. Kinetic study of conPAL3 in Fig. S5 was performed at 25 °C with 10 nM enzyme in a reaction system of 500 uL at pH 5.0 for GD10-GL and pH 7.0 for the other two Asn-containing substrates, GN10-GL and GFSYKPAYSN-GI. The concentration of peptide substrates tested ranged from 1 to 100 μM. Every 30 s, 100 μl reaction mixtures were quenched and quantitatively analyzed. Reactions for Fig. S6 were performed in triplicates with GN10-GL in nonaqueous solvents topped up with a pH 7.0 reaction buffer (20 mM sodium phosphate, 1 mM EDTA, 5 mM β-ME) at 25 °C with an E:S ratio of 1:1000. The reaction progresses were monitored by MALDI-TOF mass spectrometry analysis at 4 min, 10 min, 30 min, and 1 h.

## Data availability

All relevant data are within the article and Supporting Information files and available upon request.

## Supporting Information

This article contains supporting information. Supporting information includes 7 figures.

## Conflict of interest

The authors declare that they have no conflicts of interest with the contents of this article.
