# Supplementary material for: Consensus design and engineering of an efficient and high-yield peptide asparaginyl ligase for protein cyclization and ligation
Source: J Biol Chem. 2023 Feb 9;299(3):102997. doi: 10.1016/j.jbc.2023.102997 (PMC10017362; doi:10.1016/j.jbc.2023.102997)
Supplement: Supporting information [file mmc1.pdf]

SUPPORTING INFORMATION

Consensus design and engineering of an efficient and high-yield Peptide Asparaginyl Ligase for protein cyclization and ligation

A

conLEG expression construct

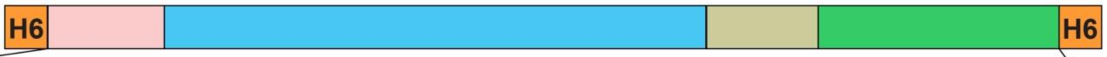


```
>cDNA_conLEG
CTGCGCCTGCCGTCTGAAGCGGCGCGTTTCTTCCGTGGCAACTCTAACGATGATGATTCCGTTGGCACCCGCTGGGCTGTGCTGATCGCGGGCTCTAACGGTTA
CTGGAACTACCGTCACCAGGCGGATGTTTGCCACGCGTACCAGCTGCTGCGTAAAGGTGGCCTGAAAGACGAAAACATCATTGTTTTTATGTACGATGATATCG
CATATAACGAAGAAAACCCGCGTCCGGGCGTGATCATCAACTCCCCGCACGGCGAAGATGTGTACAAAGGCGTTCCGAAAGATTACACCGGCGAAGATGTGACC
GTTAACAACTTCTTCGCGGTGATCCTGGGCAACAAAACCGCGCTGACCGGTGGTAGCGGTAAAGTTGTTGATTCTGGTCCGAACGATCACATCTTCATCTACTA
CTCTGATCACGGCGGACCGGGCGTGCTGGGCATGCCGACATCACCGTACCTGTACGCGGATGATCTGATCGATGTTCTGAAAAAGAAACACGCGAGCGGCACCT
ACAAAAGCCTGGTTTTCTACCTGGAAGCGTGCGAATCCGGCAGCATCTTCGAAGGTCTGCTGCCGGAAGGCCTGAACATCTACGCAACCACCGCGTCTAACGCG
GAAGAAAGCTCCTGGGGTACCTACTGCCCTGGCGAATACCCGAGCCCGCCGCCGGAATATGAAACCTGCCTGGGTGACCTGTATAGCGTTGCTTGGATGGAAGA
TAGCGATATCCACAACCTGCGTACCGAAACCCTGCATCAGCAGTACGAACTGGTTAAACGTCGTACCGCGAACGGTAACTCATATTACGGAGGCAGCCACGTTA
TGCAGTACGGTGATCTGGGTCTGAGCAAAGATAACCTGTTCCTGTACATGGGCACCAACCCGGCTAACGACAACTACACCTTCGTTGATGAAAACAGCCTGCGT
AGCCCGCTGAAAGCCGTTAACCAGCGTGATGCTGATCTGGTACACTTCTGGGATAAATACCGTAAAGCGCCGGAAGGTTCTCCGCGTAAAGTTGAAGCGCAGAA
ACAGTTCCTGGAAGCTATGAGCCATCGCATGCACATTGATAACTCCGTTAAACTGATCGGCAAACTGCTGTTCGGCATTGAAAAAGGTCCGGAAGTTCTGAACG
CGGTTCGTCCGGCGGGCCAGCCGCTGGTTGACGATTGGGACTGCCTGAAAAGCATGGTTCGTACCTTCGAAACCCATTGCGGCTCTCTGTCTCAGTACGGCATG
AAACACATGCGTAGCTTCGCTAACATCTGCAACGCGGGCATCCAGAAAGAACAGATGGGTGAAGCTAGCGCGCAGGCGTGCGTGAGCATCCCG
```

B Expression & IMAC purification **C Acid-induced activation**

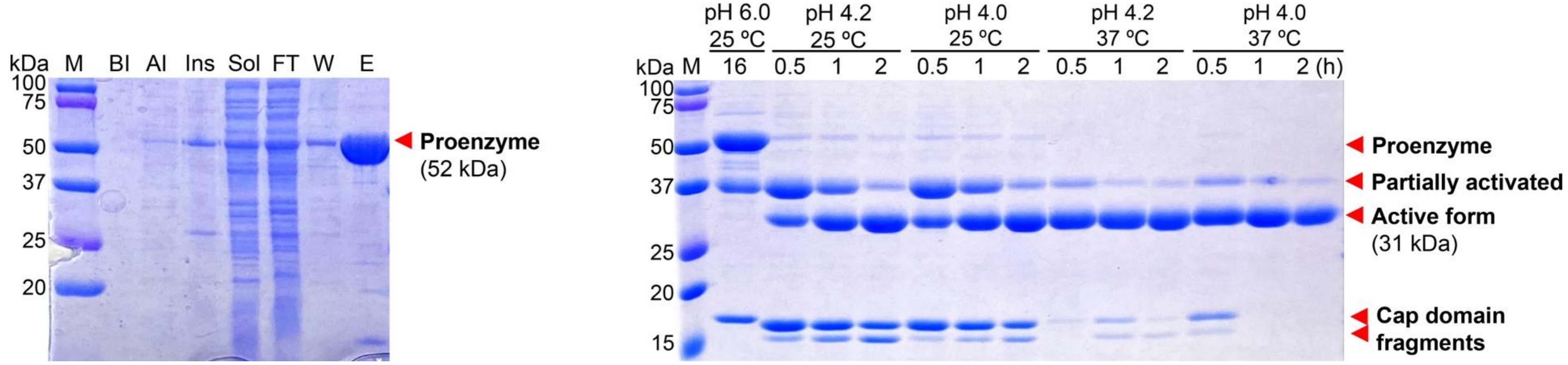

Supplementary Figure S1. **Recombinant expression and activation of conLEG.** (A) cDNA sequence for conLEG proenzyme expression construct. (B) SDS-PAGE of extraction and immobilized metal affinity chromatography (IMAC) purification. M, protein marker. BI, before induction. AI, after induction. Ins, insoluble lysate. Sol, soluble extract. FT, IMAC flow-through. W, IMAC wash-through. E, IMAC elution. (C) SDS-PAGE of activation conditions.

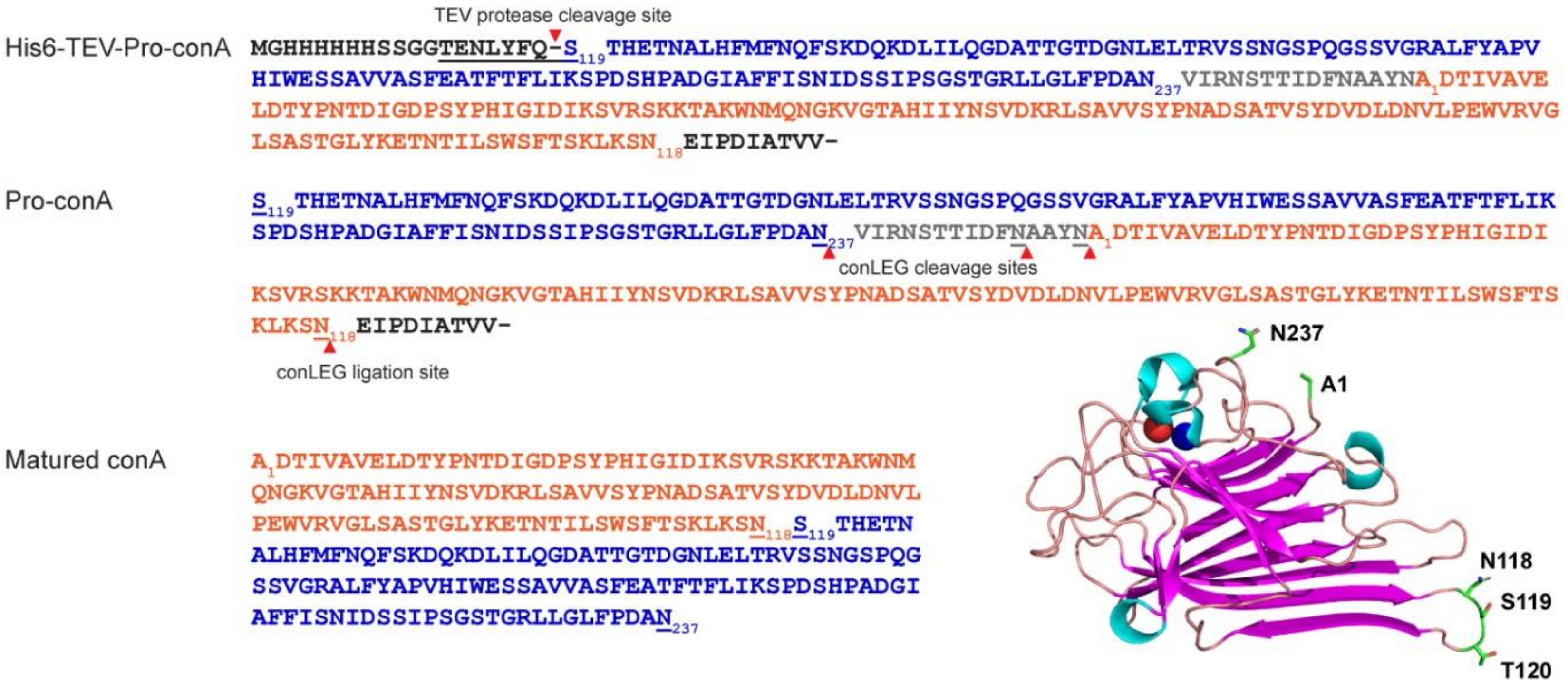

Supplementary Figure S2. Amino acid sequences of His6-TEV-Pro-conA, pro-conA precursor and matured conA. The legumain-processing sites, A1, N118, S119, N237 were labelled on the structure of a matured conA monomer (PDB ID: 1NLS).

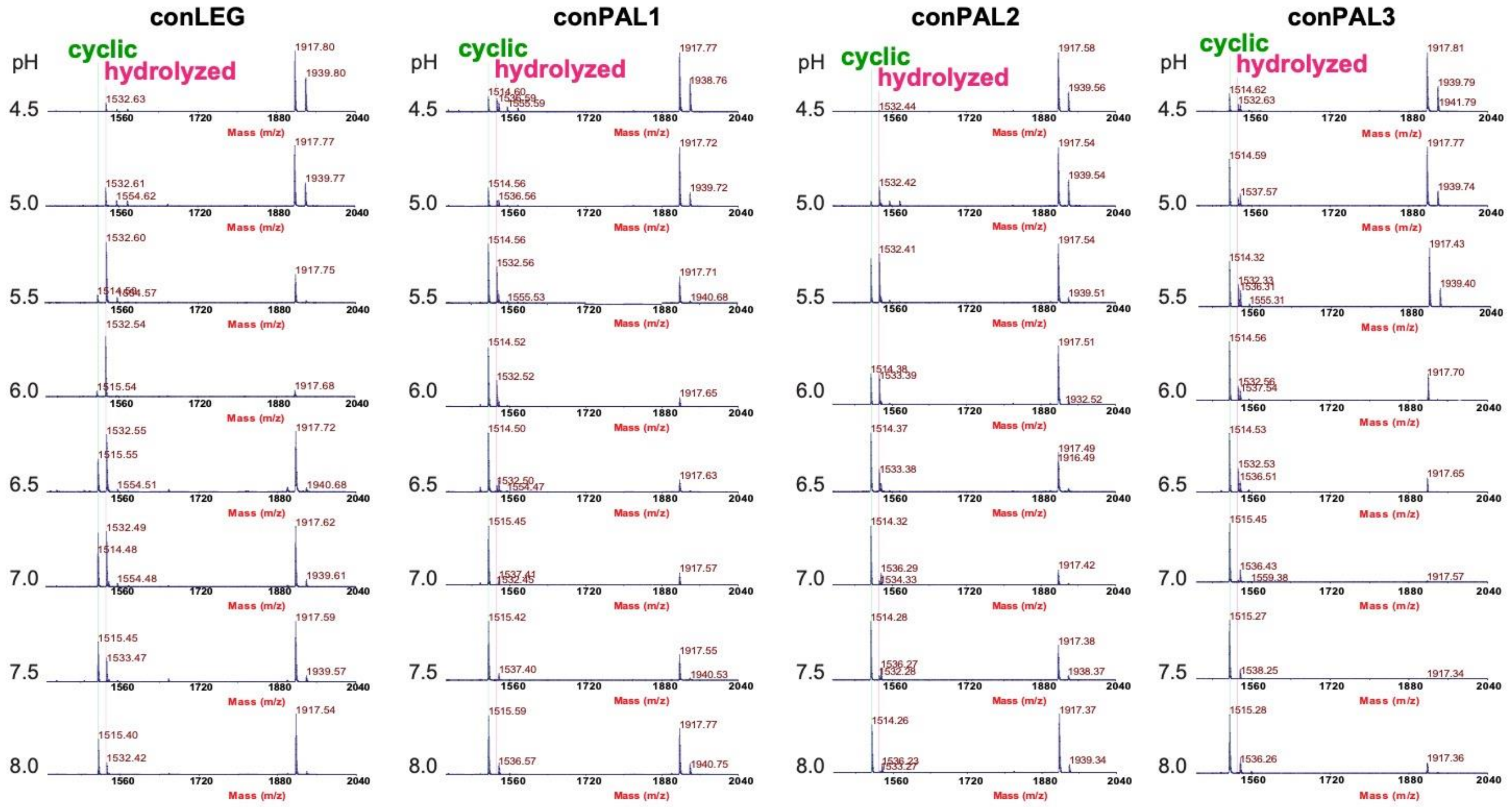

Supplementary Figure S3. **Examples of MS monitoring for conLEG1-3 activity screening.** Peptide substrate GN14-SLAN (M.W. 1916 Da) was either hydrolyzed to GN14 (M.W. 1532 Da) or cyclized to cGN14 (M.W. 1514 Da). Experiments were performed in triplicates.

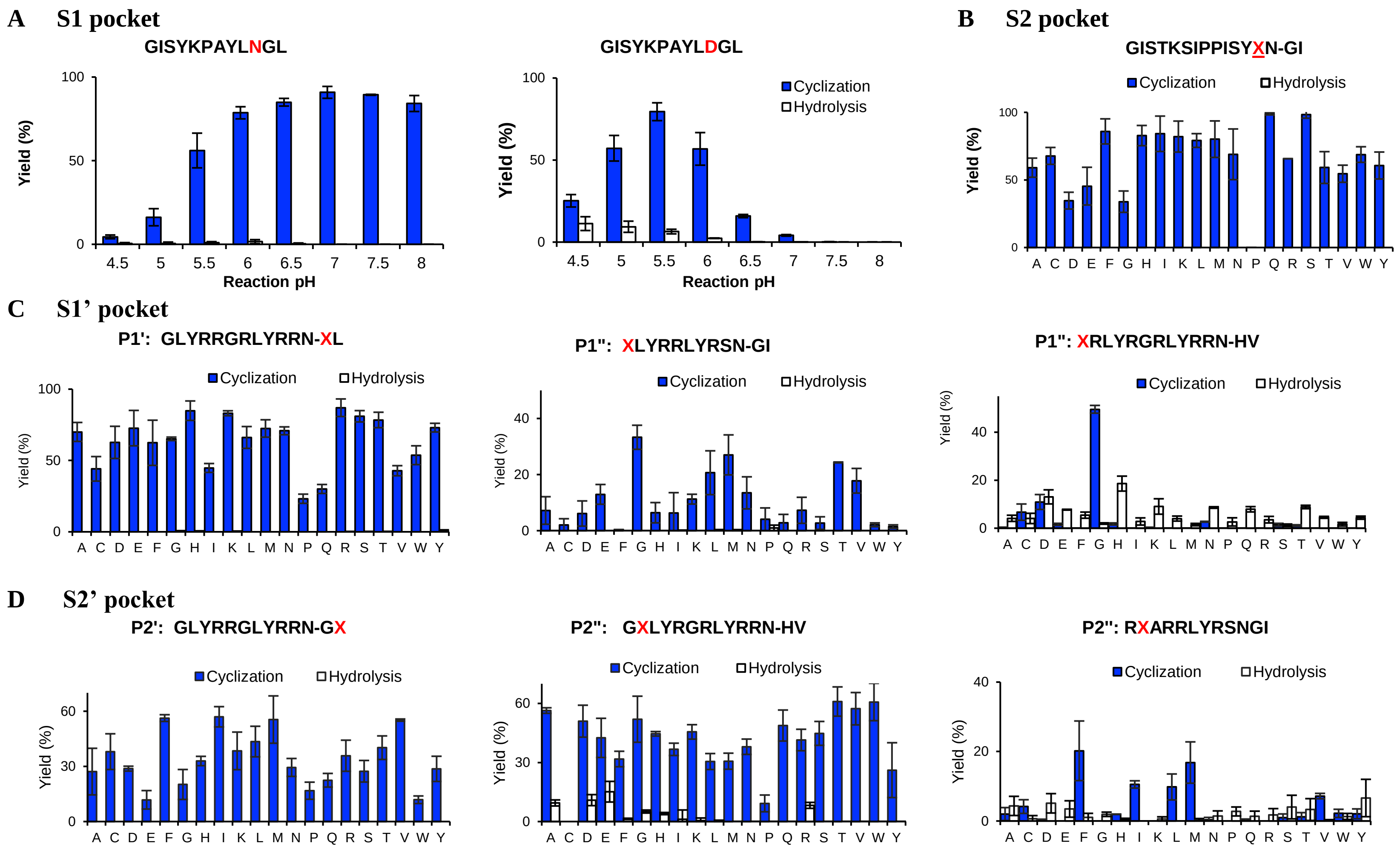

Supplementary Figure S4. Binding specificity of S2-S1-S1’-S2’ substrate pockets of conPAL3. (A) Specificity of S1 pocket against Asn and Asp. (B) Specificity study of S2 pocket shows that S2 pocket has broad tolerance to any residues except Pro. (C) Specificity study of S1’ pocket against P1’ and P1’’ residues shows that S1’ pocket is not highly selective but favor P1’’-Gly in ligation reactions. (D) Specificity study of S2’ pocket against P2’ and P2’’ residues shows that S2’ pocket favors hydrophobic residues including Phe, Ile, Leu, Met, and Val. But in the case that P1’/P1’’ is occupied by Gly, P2’ or P2’’ couold be any residues.

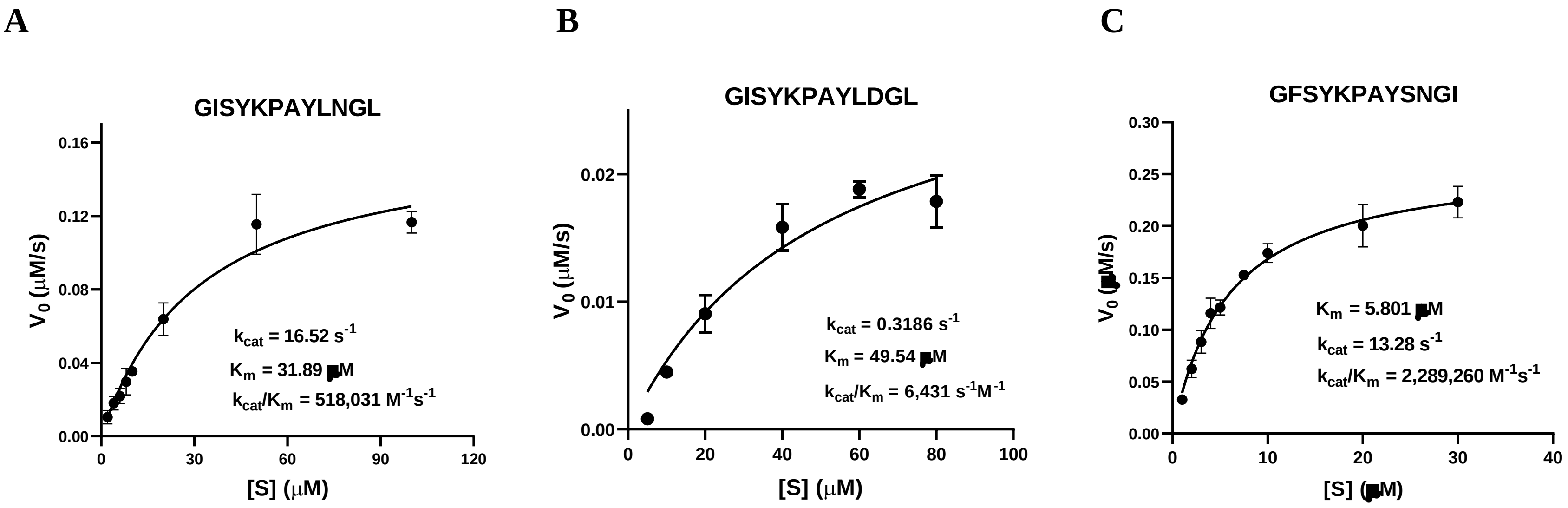

Supplementary Figure S5. Enzyme kinetic study of conPAL3 in three peptide cyclization reactions. Reactions were performed at pH 5.0 for GISYKPAYLDGL and pH 7.0 for the other two substrates at 25 °C in triplicates. Reaction mixtures were aliquoted for quantitative analysis every 30 seconds by UPLC.

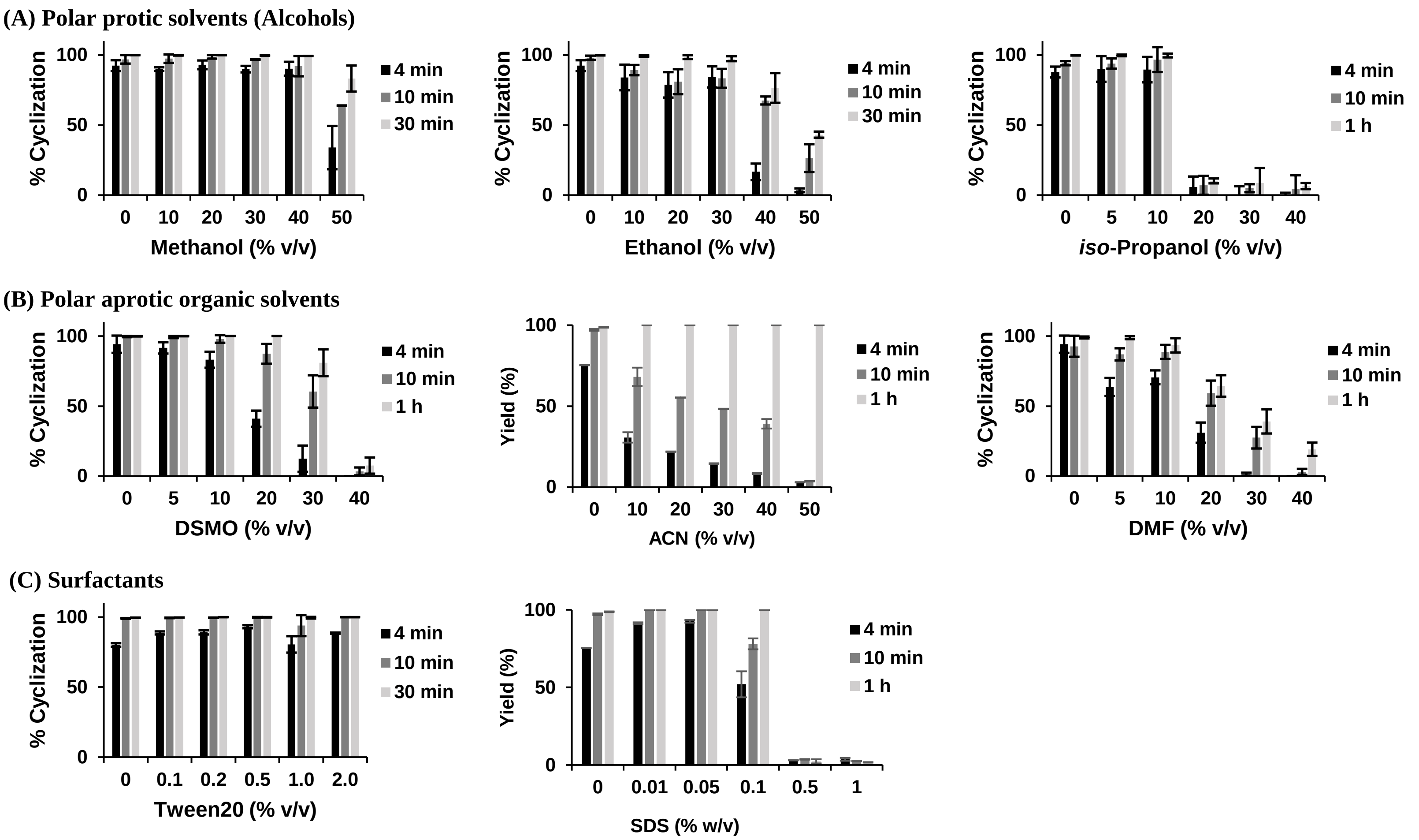

Supplementary Figure S6. Tolerance against non-aqueous solvents. (A) Polar protic solvents: methanol, ethanol, isopropanol. (B) Polar aprotic organic solvents: DMSO, ACN, DMF. (C) Surfactants: (non-ionic) Tween-20 and (anionic) SDS. Reactions were performed with GN10-GL in a mixed solution containing pH 7.0 phosphate buffer and non-aqueous solvents at 25 °C and monitored with MALDI-TOF MS.

```
PAL
Butelase1     GTRWAVLVAGSKGYVNYRHQADVCHAYQILKKGGLKDENIIVFMYDDIAYNESNPHPGVIINHPYGSDVYKGVPKDYVGEDI NPPNFYAVLLANKSALTGTGSGKVLDSGPNDHVFIYYTDHGGAGVLGMPSKPYIAASDLNDVLKKKHASGTYKSIVFYVESCESGSMFDGLLPEDHNI   180
OaAEP1b       GTRWAVLIAGSKGYANYRHQAGVCHAYQILKRGGLKDENIVVFMYDDIAYNESNPRPGVIINSPHGSDVYAGVPKDYTGEEV NAKNFLAAILGNKSAITG-GSGKVVDSGPNDHIFIYYTDHGAAGVIGMPSKPYLYADELNDALKKKHASGTYKSLVFYLEACESGSMFEGILPEDLNI   179
OaAEP3        GTRWAVLIAGSKGYDNYRHQADVCHAYQILKRGGLKDENIVVFMYDDIAYNESNPRPGVIINSPHGSDVYAGVPKDYTGDEV NAKNFLAAILGNKSAITG-GSGKVVDSGPNDHIFIYYTDHGAPGVIGMPSKPYLYADELNDALRKKHASGTYKSMVFYLEACEAGSMFDGLLPDGLNI   179
OaAEP4        GTRWAVLLAGSNGYWNYRHQADLCHAYQILKRGGLKDENIVVFMYDDIAYNEENPRPGVIINSPHGSDVYAGVPKDYTGDEV NAKNFLAAILGNKSAITG-GSGKVVDSGPNDHIFIYYTDHGAPGVIGMPSKPYLYADELNDALRKKHASGTYKSMVFYLEACEAGSMFDGLLPDGLNI   179
OaAEP5        GTRWAVLIAGSKGYDNYRHQADVCHAYQILKRGGLKDENIVVFMYDDIAYNESNPRPGVIINSPHGSDVYAGVPKDYTGEEV NAKNFLAAILGNKSAITG-GSGKVVDSGPNDHIFIYYTDHGAAGVIGMPSKPYLYADELNDALKKKHASGTYKSLVFYLEACEAGSMFEGLLTDDLNI   179
HeAEP3        GTRWAVLIAGSKGWQNYRHQADVCHAYQILKKGGLKDENIIVFMYDDIAYNESNPRPGIVINKPKGEDVYKGVPKDYTGENV NAVNFLAVLLANRSALTG-GSGKVLDSGPNDRIFIYYTDHGAPVTIGMPSKPYLVAKDLVDTLKKKHAAGTYKSMVFYIESCESGSMFDGLLPEDANI   179
VyPAL1        GTRWAVLIAGSKGYHNYRHQADVCHMYQILRKGGVKDENIIVFMYDDIAYNESNPFPGIIINKPGGENVYKGVPKDYTGEDI NNVNFLAAILGNKSAIIG-GSGKVLDTSPNDHIFIYYADHGAPGKIGMPSKPYLYADDLVDTLKQKAAAGTYKSMVFYVEACNAGSMFEGLLPEGMNI   179
VyPAL2        GTRWAVLIAGSKGYHNYRHQADVCHMYQILRKGGVKDENIIVFMYDDIAYNESNPFPGIIINKPGGENVYKGVPKDYTGEDI NNVNFLAAILGNKSAIIG-GSGKVLDTSPNDHIFIYYADHGAPGKIGMPSKPYLYADDLVDTLKQKAATGTYKSMVFYVEACNAGSMFEGLLPEGTNI   179
VyPAL4        GTTWAVLIAGSKGYHNYRHQADVCHMYQILRKGGVKDENIIVFMYDDIAYNESNPFPGIIINKPGGENVYKGVPKDYTGEDI NNVNFLAAILGNKSAIIG-GSGKVLDTSPNDHIFIYYADHGAPGKIGMPSKPYLYADDLVDTLKQKAAAGTYKSMVFYVEACNAGSMFEGLLPEGMNI   179
VyPAL5        GTTWAVLIAGSKGYHNYRHQADVCHMYQILRKGGVKDENIIVFMYDDIAYNESNPFPGIIINKPGGENVYKGVPKDYTGEDI NNVNFLAAILGNKSAIIG-GSGKVLDTSPNDHIFIYYADHGAPGKIGMPSKPYLYADDLVDTLKQKAATGTYKSMVFYVEACNAGSMFEGLLPEGTNI   179

AEP
Butelase2     GTRWAVLVAGSNGYENYRHQADVCHAYQLLIKGGLKEENIVVFMYDDIAWHELNPRPGVIINNPRGEDVYAGVPKDYTGEDV TAENLFAVILGDRSKVKG-GSGKVINSKPEDRIFIFYSDHGGPGVLGMPNEQILYAMDFIDVLKKKHASGGYREMVIYVEACESGSLFEGIMPKDLNV   179
VyAEP1        GTRWAVLVAGSNGFGNYRHQADVCHAYQLLRKGGLKEENIVVFMYDDIATNQLNPRPGIIINHPQGEDVYHGVPKDYTGAEV NAHNLYAVLLGDKSAVKG-GSGKVVNSKPDDRIFVYYSDHGGPGVLGMPNLPYVYAMDFIDTLKKKHAANSYREMVVYVEACESGSLFEGVMPKDLNI   179
OaAEP2        GTRWAVLLAGSNGYWNYRHQADLCHAYQILKRGGLKDENIVVFMYDDIAYNEENPRPGVIINSPHGSDVYAGVPKDYTGDQV NAKNFLAAILGNKSAITG-GSGKVVNSGPNDHIFIYYTDHGGPGVLGMPVGPYIYADDLIDTLKKKHASGTYKSLVFYLEACESGSMFEGLLPEGLNI   179
CeAEP         GTRWAVLVAGSNGYGNYRHQADVCHAYQLLIKGGVKEENIVVFMYDDIAYNAMNPRPGVIINHPQGPDVYAGVPKDYTGEDV TPENLYAVILGDKSKVKG-GSGKVINSNPEDRIFIFYSDHGGPGVLGMPNAPFVYAMDFIDVLKKKHASGGYKEMVIYIEACESGSIFEGIMPKDLNI   179
RcAEP         GTRWAVLVAGSMGFGNYRHQADVCHAYQLLRKGGLKEENIIVFMYDDIAKNELNPRPGVIINHPQGEDVYAGVPKDYTGEHV TAKNLYAVLLGDKSAVQG-GSGKVVDSKPNDRIFLYYSDHGGPGVLGMPNLPYLYAMDFIEVLKKKHAAGGYKKMVIYVEACESGSIFEGIMPKDVDI   179
HaAEP         GTKWAVLVAGSKGYGNYRHQADVCHAYQVLKKGGLKDENIVVFMYDDIAKSEMNPRPGIIINSPKGEDVYAGVPKDYTGKNV TVDNLSAVLLGDRSAVKG-GSGKVVDSKPEDRIFLFYSDHGGPGVLGMPNEPHLVAKDLVDVLKKKHAMGTYKEMVIYLEACESGSIFEGILPEDLNI   179
AtVPE-alpha   STRWAVLVAGSSGYWNYRHQADVCHAYQLLKKGGVKEENIVVFMYDDIAKNEENPRPGVIINSPNGEDVYNGVPKDYTGDDV NVDNLLAVILGNKTAVKG-GSGKVVDSGPNDHIFIYYSDHGGPGVLGMPTSPYLYANDLNDVLKKKHASGTYKSLVFYLEACESGSIFEGLLPEGLNI   179
AtVPE-beta    GTRWAVLVAGSSGYGNYRHQADVCHAYQILRKGGLKEENIVVLMYDDIANHPLNPRPGTLINHPDGDDVYAGVPKDYTGSSV TAANFYAVLLGDQKAVKG-GSGKVIASKPNDHIFVYYADHGGPGVLGMPNTPHIYAADFIETLKKKHASGTYKEMVIYVEACESGSIFEGIMPKDLNI   179
AtVPE-gamma   GTRWAVLVAGSSGYWNYRHQADICHAYQLLRKGGLKEENIVVFMYDDIANNYENPRPGTIINSPHGKDVYQGVPKDYTGDDV NVDNLFAVILGDKTAVKG-GSGKVVDSGPNDHIFIFYSDHGGPGVLGMPTSPYLYANDLNDVLKKKHALGTYKSLVFYLEACESGSIFEGLLPEGLNI   179
AtVPE-delta   GTRWAVLVAGSNEYYNYRHQADICHAYQILRKGGLKDENIIVFMYDDIAFSSENPRPGVIINKPDGEDVYKGVPKDYTKEAV NVQNFYNVLLGNESGVTG-GNGKVVKSGPNDNIFIYYADHGAPGLIAMPTGDEVMAKDFNEVLEKMHKRKKYNKMVIYVEACESGSMFEGILKKNLNI   179

conLEG        GTRWAVLIAGSNGYWNYRHQADVCHAYQLLRKGGLKDENIIVFMYDDIAYNEENPRPGVIINSPHGEDVYKGVPKDYTGEDV TVNNFFAVILGNKTALTG-GSGKVVDSGPNDHIFIYYSDHGGPGVLGMPTSPYLYADDLIDVLKKKHASGTYKSLVFYLEACESGSIFEGLLPEGLNI   179
              .* ****:***  : ******.:** **:* :**:*:***:*:******    ** ** :** * * :** ******.   : .  *:  .:*.:.. : * *.***: : *:*.:*::*:***.   :.**    : * :: :.*.:      *..:*.*:*:*::**:*:*:: .. ::

                                                                                                                   %Identity(conLEG)
PAL
Butelase1     YVMGASDTGESSWVTYCPLQHPSPPP--EYDVCVGDLFSVAWLEDCDVHNLQTETFQQQYEVVKNKTIVAL-IEDGTHVVQY GDVGLSKQTLFVYMGTDPANDN   281 | 73.48
OaAEP1b       YALTSTNTTESSWCYYCPAQEN-PPP-PEYNVCLGDLFSVAWLEDSDVQNSWYETLNQQYHHVDKRIS------HASHATQY GNLKLGEEGLFVYMGSNPANDN   275 | 74.55
OaAEP3        YALTASNTTEGSWCYYCPGQ-DAGPP-PEYSVCLGDFFSIAWLEDSDVHNLRSETLNQQYHNVKNRISY------ASHATQY GDLKRGVEGLFLYLGSNPENDN   275 | 75.55
OaAEP4        YALTASNTTEGSWCYYCPGQ-DAGPP-PEYSVCLGDFFSIAWLEDSDVHNLRSETLNQQYHNVKNRISY------ASHATQY GDLKRGVEGLFLYLGSNPENDN   275 | 75.91
OaAEP5        YALTASNATEGSCPYYCPGDLNYSPP-PEYDVCLGDFFSIAWLEDSDVHNLRSETLNQQYHNVKNRISY------ASHATQY GDLKRGVEGLFLYLGSNPENDN   276 | 76.00
HeAEP3        YGMTATNSTEGSWVTYCPGQTDDYPEDDEYDVCFGDLWSVAWLEDCDAHNLRTETLDQQYEVVKKKIEY------AHIPAQY GNVSLAKDSLFVYMGTDPANDN   277 | 71.27
VyPAL1        YAMTASNSTEGSLIAYCAGVTPGVPL--EIVTCLGDLWSITFLEDCDAHNLRTETVHQQFELVKKKIAY------ASTVSQY GDIPISKDSLSVYMGTDPANDN   275 | 71.90
VyPAL2        YAMAASNSTEGSWITYCPGT-PDFPP--EFDVCLGDLWSITFLEDCDAHNLRTETVHQQFELVKKKIAY------ASTVSQY GDIPISKDSLSVYMGTDPANDN   274 | 72.89
VyPAL4        YAMTASNSTEGSLIAYCAGVTPGVPL--EIVTCLGDLWSITFLEDCDAHNLRTETVHQQFELVKKKIAY------ASTVSQY GDIPISKDSLSVYMGTDPANDN   275 | 71.53
VyPAL5        YAMAASNSTEGSWITYCPGT-PDFPP--EFDVCLGDLWSITFLEDCDAHNLRTETVHQQFELVKKKIAY------ASTVSQY GDIPISKDSLSVYMGTDPANDN   274 | 72.53

AEP
Butelase2     FVTTASNAQENSWGTYCPGTEPSPPP--EYTTCLGDLYSVAWMEDSESHNLRRETVNQQYRSVKERTSNFKDYAMGSHVMQY GDTNITAEKLYLFQGFDPATVN   281 | 71.33
VyAEP1        YVTTASNAQENSWGTYCPGEG--APP--EYNTCLGDLYSVAWMEDSESHNLKKEAIKDQYKTVKARTSDSSTYHSGSHVMEY GNRSIRAEKLYLYQGFDPATVN   279 | 68.71
OaAEP2        YATTASNAEESSWGTYCPGEYP-SPP-PEYDTCLGDLYSVAWMEDSEVHNLRSETLKQQYHLVKARTSNGN-SAYGSHVMQY GDLKLSVDNLFLYMGTNPANDN   280 | 87.50
CeAEP         YVTTASNAQENSFGTYCPGMNPPPPE--EYVTCLGDLYSVSWMEDSETHNLKRETVQQQYQSVRKRTSNSNSYRFGSHVMQY GDTNITAEKLYLYHGFDPATVN   281 | 71.33
RcAEP         YVTTASNAQESSWGTYCPGMEPSPPP--EFTTCLGDLYSVAWMEDSESHNLKKETVKQQYSSVKARTSNYNTYAAGSHVMQY GNQSIKADKLYLFQGFDPASVN   281 | 73.12
HaAEP         YATTASGAQENSYGTYCPGTEPSPPP--EYITCLGDLYSVAWMEDSETHNLKKESLEQQFNKVKKRTSNSNTYNTGSHVMEY GSKDIKPEKVYLYLGFDPATVN   281 | 71.33
AtVPE-alpha   YATTASNAVESSWGTYCPGEDP-SPP-SEYETCLGDLYSVAWMEDSDIHNLQTETLHQQYELVKKRTAGSG-KSFGSHVMEF GDIGLSKEKLVLYMGTNPANEN   280 | 86.79
AtVPE-beta    YVTTASNAQESSYGTYCPGMNPSPPS--EYITCLGDLYSVAWMEDSETHNLKKETIKQQYHTVKMRTSNYNTYSGGSHVMEY GNNSIKSEKLYLYQGFDPATVN   281 | 69.18
AtVPE-gamma   YATTASNAEESSWGTYCPGEEP-SPP-PEYETCLGDLYSVAWMEDSGMHNLQTETLHQQYELVKRRTAPVG-YSYGSHVMQY GDVGISKDNLDLYMGTNPANDN   280 | 88.21
AtVPE-delta   YAVTAANSKESSWGVYCPESYPPPPS--EIGTCLGDTFSISWLEDSDLHDMSKETLEQQYHVVKRRVGSD--VPETSHVCRF GTEKMLKDYLSSYIGRNPENDN   279 | 64.03

conLEG        YATTASNAEESSWGTYCPGEYP-SPP-PEYETCLGDLYSVAWMEDSDIHNLRTETLHQQYELVKRRTANGN-SYYGSHVMQY GDLGLSKDNLFLYMGTNPANDN   280
              :   ::.: *.*   **       *   *  .*.** :*::::**.  ::   *:..:*:  *  :              .: *      : :  : * :* . *
```

Supplementary Figure S7. Amino acid sequence alignment and percentage of sequence identity between conLEG and 20 other plant legumains.
